# Supplementary material for: Prognostic Significance of Programmed Cell Death Ligand 1 Expression in High-Grade Serous Ovarian Carcinoma: A Systematic Review and Meta-Analysis
Source: Diagnostics (Basel). 2023 Oct 19;13(20):3258. doi: 10.3390/diagnostics13203258 (PMC10606661; doi:10.3390/diagnostics13203258)
Supplement: Supplementary file 1 [file diagnostics-13-03258-s001.zip › diagnostics-2656203-supplementary.pdf]

Table S1. New Castle Ottawa Scale for Case-control studies

	Selection				Comparability	Exposure			Score
Study	Is the case definition adequate?	Representativeness of the cases	Selection of Controls	Definition of Controls	Comparability of cases and controls on the basis of the design or analysis	Ascertainment of exposure	Same method of ascertainment for cases and controls	Non-Response rate	
Aust 2017 [10]	★	★		★	★★	★	★		7
Bansal 2021 [11]	★	★		★	★★	★	★		7
Bas 2021 [12]	★			★	★★	★	★		6
Chen 2020 [13]	★	★		★	★★	★	★		7
Darb-Esfahani 2015 [14]	★			★	★★	★	★		6
de la Fuente 2020 [22]	★	★		★	★★	★	★		7
Eymerit-Morin 2021 [15]	★	★		★	★★	★	★		7
Farrag 2021 [16]	★	★		★	★★	★	★		7
Fucikova 2019 [17]	★	★		★	★★	★	★		7
Henriksen 2020 [18]	★	★		★	★★	★	★		7
Kim 2018 [19]	★	★		★	★★	★	★		7
Lee 2022 [20]	★	★		★	★★	★	★		7
Li 2017 [21]	★	★		★	★★	★	★		7
Mills 2019 [23]	★	★		★	★★	★	★		7
Wang 2017 [24]	★	★		★	★★	★	★		7
Wieser 2018 [49]	★	★		★	★★	★	★		7
Zhang 2023 [25]	★	★		★	★★	★	★		7
